# Skin-Grafting

**Published:** 1889-03

**Authors:** 


### Skin-Grafting.

To the Editor.

*Sir:*—To an observer of the literature of the progress of practical surgery it is curious to see how frequently old, discarded practices are revived and proclaimed as novelties. Your recent editorial on "Transplantation of Skin from a Corpse" is a marked illustration of such a useless resurrection of a long defunct subject.

Many years ago, soon after the success of the process of skin grafting was anounced, I tried grafting from the integument of recently amputated limbs, and also from the cadaver. The success of these grafts was quite equal to that from the living subject. But I soon discontinued the practice of grafting from the cadaver and from amputated limbs, and from any source but the patient's own integument, and publicly deprecated and condemned it as subjecting the patient to serious risks of specific infection.

You need not be troubled by the fear that the recent revival of this improper operation by a German surgeon "is likely to imperil the claim of our country for having originated it." I believe that I was the first who experimentally performed it, but would prefer to make the claim of having been the first to discard and condemn it.

I have successfully transplanted the skin of a rabbit to a raw surface that was denuded in a plastic operation on the face. In a noted case of cicatricial deformity of the face I successfully formed an entire upper eyelid from the integument of an anchylosed and useless finger that I had previously amputated from the patient's own hand.

I send to you herewith a copy of my pamphlet on skin grafting, which was originally published in the *Philadelphia Medical Times* about twelve years ago.

Yours truly,

Richard J. Levis, M.D.

Philadelphia, Sept. 25, 1888.

—*Medical and Surgical Journal.*

A Beautiful and Durable Cement for Ringing Balsam Mounts.—Mr. J. D. Beck sends the following to the *Microscope*, Jan., 1889: To a thick solution of gum arabic add a little glycerine to prevent cracking. Ring balsam mounts with this first, then finish with the same cement colored with magenta, or fuchsine, or the "Diamond" black dye dissolved in water. Ornament with gold paint, etc., and finish with "Winsor & Newton's" mastic picture varnish. Try cement on a blank slide; if brittle when hard, add a little more glycerine, so that it will harden in twenty-four hours without brittleness.

---

Jonathan Hutchinson, of London, many years ago observed that, coexisting with this disease, there is also frequently present in the young subjects of heredity syphilis a peculiar coloring and conformation of the teeth, more especially of the two upper permanent central incisors. The peculiarity consists in a dirty brownish coloration, together with a dwarfing, or narrowing and shortening of these teeth, with a gouged-out condition of the central portion of the cutting end, so that a pegged appearance of the two teeth results. At one time he thought that, in a well-marked case, such teeth alone were sufficient to prove the patient to be syphilitic. The observation was a praiseworthy one, but the conclusion is too sweeping. In many cases there would seem to be a strange coincidence to say the least, of the three conditions—syphilis, diffuse parenchymatous keratitis, and Hutchinson's teeth. Yet in our little patient the pegged teeth are not present. About the seventh year the permanent incisors appear, but this child is in her eleventh year, and the upper central incisors are not yet fully grown. Their color is white, the teeth are broad and their cutting edges are serrated instead of pegged —none of which conditions is characteristic of Hutchinson's teeth; and yet we have a positive history of hereditary syphilis.

The late appearance of the teeth may be attributed to general malnutrition, and the latter is certainly due to the specific disease.

---

A dental school is to be established in connection with the University of Vienna.

ABUSE OF NEW DRUGS—The abuse of new drugs, says the *Kansas City Medical Record*, is a rapidly increasing evil. All along the line, from the metropolitan to the cross-roads doctors, the tendency is a rush for fame by being the first to employ a new drug for a new purpose, and after one or two trials, with probably imaginary success, they leap for the medical press like a lot of small boys running to a fire. Many physicians in recent years have even contracted the habit of hypothecating in order to give their statements more tone. Among the more abused drugs are antipyrin and antifebrine. They are recommended for the cure of every thing except ingrowing toenails. There is but one safe way of testing the action of any remedy, and that is by collective investigation and comparison, and this can be more carefully done in large hospitals.

